# Case Report: Improving Verbal Retrieval Deficits With High Definition Transcranial Direct Current Stimulation Targeting the Pre-Supplementary Motor Area in a Patient With Chronic Traumatic Brain Injury

**DOI:** 10.3389/fneur.2021.678518

**Published:** 2021-07-16

**Authors:** Hsueh-Sheng Chiang, Scott Shakal, Sven Vanneste, Michael Kraut, John Hart

**Affiliations:** ^1^Department of Neurology, The University of Texas Southwestern Medical Center, Dallas, TX, United States; ^2^School of Behavioral and Brain Sciences, The University of Texas at Dallas, Richardson, TX, United States; ^3^Texas College of Osteopathic Medicine, The University of North Texas Health Science Center, Fort Worth, TX, United States; ^4^Trinity College Dublin, Dublin, Ireland; ^5^Department of Radiology and Radiological Science, The Johns Hopkins University School of Medicine, Baltimore, MD, United States

**Keywords:** TBI, verbal retrieval, HD-tDCS, EEG, case report, verbal fluency, tDCS, word finding

## Abstract

We report a patient who has cognitive sequalae including verbal retrieval deficits after severe traumatic brain injury (TBI). The cortico-caudate-thalamic circuit involving the pre-Supplementary Motor Area (pre-SMA) has been proposed to underlie verbal retrieval functions. We hypothesized that High Definition-transcranial Direct Current Stimulation (HD-tDCS) targeting the pre-SMA would selectively modulate this circuit to remediate verbal retrieval deficits. After the patient underwent 10 sessions of 20 min of 1 mA HD-tDCS targeting the pre-SMA, we documented significant improvements for verbal fluency and naming, and for working memory and executive function tasks that involve the frontal lobes. The effects persisted for up to 14 weeks after completion of HD-tDCS treatment. We also demonstrated normalization of the event-related potentials suggesting modulation of the underlying neural circuit. Our study implicates that region-specific non-invasive brain stimulation, such as HD-tDCS, serves as a potential individualized therapeutic tool to treat cognitive deficits by inducing longer-lasting neuroplasticity even in the chronic phase of TBI.

## Introduction

Traumatic brain injury (TBI) can result in a variety of deficits including cognitive, neurological, and emotional dysfunction (1). There are few if any standardized treatments for the cognitive sequela of TBI, including word finding difficulties which are among the most frequently reported (2–5). These word finding difficulties may reflect focal injury to specific brain regions and/or diffuse injury that disrupts connections between the regions that subserve word retrieval (6). Based on a series of neuroimaging and neuropsychological studies, Hart et al. (7, 8) have proposed a neural circuit involving the pre-Supplementary Motor Area (pre-SMA), caudate, and thalamus that mediates verbal retrieval functions essential to semantic and episodic memory. In this model, the pre-SMA serves as an essential hub for memory and verbal retrieval, involved in the initiation and selection processes during retrieval. Lesions located in the pre-SMA and its vicinity have been associated with deficits in memory retrieval and word production, in particularly during volitional language-based and motor-based response selection (9–12).

Non-invasive brain stimulation has been used to test theories of neurocognitive constructs as well as to modulate neural functions to enhance cognition. We focus on transcranial Direct Current Stimulation (tDCS), which leads to modulation of cortical excitability by biasing membrane potentials toward polarization at a subthreshold level and has been tested in TBI populations with promising results (13–15). Spatially, tDCS affects both the superficial cortical structures immediately subjacent to the stimulating electrode as well as deep brain structures (16, 17). Although repetitive application may lead to more persistent behavioral changes and has been linked to various possible changes in neurotransmitter receptors, ion channels, synaptic potentiation/depression based on both *in vitro* and *in vivo* studies, with the detailed mechanisms awaiting further clarification (18, 19).

Here we report an individual with cognitive sequalae of TBI, including a significant deficit in verbal fluency and naming. We applied High Definition tDCS (HD-tDCS) (20, 21) that entails better focality than conventional tDCS in order to selectively target the pre-SMA and thus to modulate the pre-SMA-caudate-thalamic circuit underlying verbal retrieval deficits. We documented significant therapeutic effects using longitudinal neuropsychological testing corroborated by neurophysiological measures (cognitive task-related event-related potentials) to investigate potential underlying neural mechanisms.

## Single Case Description

**Figure 1:**
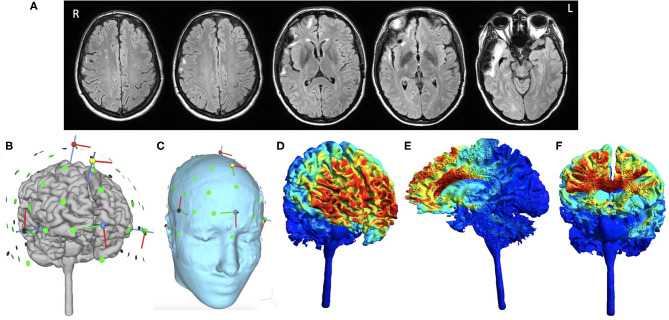
Baseline MRI brain images and HD-tDCS montage. Selected axial T2-weighted/FLAIR images that depict encephalomalacia involving the right frontal and temporal lobes and several foci of signal hyperintensity within the right frontal centrum semiovale **(A)**. HD-tDCS is applied using a 4 × 1 montage, including the anodal electrode at Fz (1 mA) paired with four cathodal electrodes at FPz, Cz, F7, F8 (0.25 mA each) as shown after reconstruction of the subject's gray matter surface **(B)** and scalp **(C)**. Normalized electric field is then simulated to represent its intensity and distribution based on the subject's own T1 and T2 weighted images presented over the gray matter surface **(D)**, saggital section **(E)**, and coronal section **(F)**. Even though the distribution of electric field is slightly asymmetric due to chronic right temporal lobe volume loss and right lateral ventricular enlargement, the focus of HD-tDCS is located to the medial superior part of the pre-Supplementary area and the dorsal anterior cingulate cortex. Normalized electric field is in the unit of Volt per meter. All analyses were performed using SimNIBS 3.2.1 (22).

A 39-year-old right-handed (dominant hand), Caucasian female was referred to our memory clinic 3 years after a bicycle accident, which resulted in a left temporo-parietal epidural hematoma and scattered right temporal lobe contusions that required surgical evacuation and intensive care unit monitoring. Retrospectively, post-traumatic amnesia was unclear and loss of consciousness was prolonged. This would be most appropriately graded as severe TBI although we do not have information on her initial Glasgow Coma Scale and length of hospitalization. Brain MRI acquired within a few months of treatment initiation showed encephalomalacia involving the right frontal and temporal lobes (Figure 1A). Even though there was no evident encephalomalacia near the previous left epidural hematoma, there was visibly reduced volume in the left parietal region suggesting prior injury. She had significant functional recovery and regained her independence in daily functioning. Modified Rankin Scale was 2 at post-TBI baseline. Prior to the injury, she was a high functioning executive with 18 years of education. She was not able to resume her prior job as a manager due to persistent cognitive complaints and frequent episodes of dizziness. She was taking levetiracetam 500 mg twice daily due to a post-TBI seizure immediately after the accident and she had been seizure free since then. She had a history of migraine but did not have any pre-morbid developmental delay or learning disabilities. Of note, during the study she was on a stable dose of off-label use of ropinirole 0.75 mg twice daily for at least 3–4 months to treat her verbal retrieval deficits, with reported mild improvement. Use of dopaminergic agents for improving verbal fluency has been reported in the literature with mixed results [see review by (23) for its potential mechanisms], as we have observed in our clinical practice with often variable success among different individuals.

### Description of Reported Cognitive Symptoms

The patient reported stable, persistent cognitive symptoms for at least the past 2 years, including difficulties with concentration to complete a task, word finding, naming objects, understanding what she has read, difficulties in remembering names and trouble recognizing people's faces, organizing ideas and shifting attention. She denied any such issues prior to the accident.

## Experimental Assessment and Therapeutic Intervention

The patient was recruited as part of an open-label group study using HD-tDCS targeting the pre-SMA for cognitive deficits in patients with chronic TBI. Both safety and feasibility were evaluated in this study. HD-tDCS (with a similar 4 × 1 montage) has been tested extensively in healthy subjects, with side effects reported to be minimal even at a current significantly higher than ours (24). However, there is lack of large-scale studies that systematically examine safety in neurologic patients, other than case reports and case series. We are not aware of studies reporting major safety concerns for HD-tDCS application in neurologic patients that precluded our study protocol. We strictly followed exclusion criteria for tDCS protocols [e.g., presence of electronic implants or any extracranial or intracranial foreign objects (25)] and closely executed safety protocol including having a trained physician on site, and closely monitoring the patient's symptoms during and after HD-tDCS sessions. Informed consent was obtained in accordance with the protocols approved by the Institutional Review Board of the University of Texas at Dallas and the University of Texas Southwestern Medical Center.

### HD-tDCS Protocol and Outcome Measures

The HD-tDCS montage targeting the pre-SMA consisted of Fz for the central anodal electrode and FPz, Cz, F7, and F8 for the return (cathodal) electrodes (i.e., five circular Ag/AgCl electrodes 1 cm radius with conductive gel). A battery-driven, wireless multichannel transcranial current source generated the stimulation current (Neuroelectrics Starstim®). This montage has been reported in previous studies (15, 21) and was designed to target the dorsomedial prefrontal cortex, with the electric field most concentrated in the pre-SMA/dACC based on electric field simulation (Figures 1B–F; including pre-SMA and part of the dorsal Anterior Cingulate Cortex, dACC). At each session, active HD-tDCS was ramped up over 60 s until it reached 1 mA, maintained at 1 mA for 20 min, and then ramped down to 0 mA over 60 s. This current was selected based on a prior study that showed good tolerability in healthy adults (21). HD-tDCS was administered across 10 sessions over 2 weeks, each constituting 20 min of stimulation while the patient sat quietly or engaged in casual conversation with the experimenter. We assessed the patient's pain and comfort level during and after each session. The patient only experienced minimal tingling sensation near the electrodes in the beginning of each session, and did not report discomfort or any other side effects.

**Figure 2:**
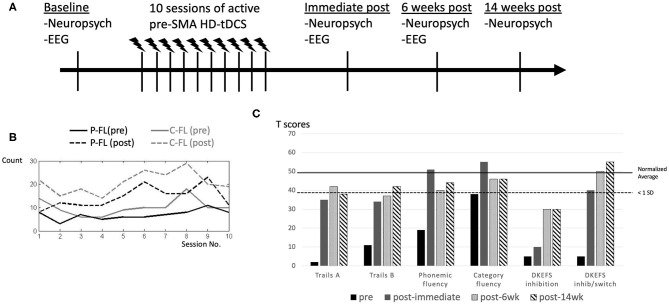
Study protocol and performance change in response to HD-tDCS after each session and at longitudinal follow ups after completion of intervention. Study protocol is demonstrated **(A)**. Verbal fluency demonstrated significant improvements after each single HD-tDCS session, which wore off prior to the next session, but the post-session performance had a steady improvement as the number of sessions increased, with some expected fluctuations **(B)**. Phonemic fluency (P-FL) was scored with the summed number of items from COWAT (letters F, A, and S) and category fluency (C-FL) was scored with the total item using animal category **(B)**. At baseline, category fluency was more than 1 standard deviation (SD) below average. Performance on most of these tests improved to closer to or within 1 standard deviation from average after the HD-tDCS intervention, except for D-KEFS inhibition. These positive effects persisted over 6 and 14 weeks **(C)**. References for these neuropsychological measures are cited in Table 1.

The overall study protocol is demonstrated in Figure 2A. We administered verbal fluency (phonemic and category) before and after each single HD-tDCS session in order to monitor instantaneous and cumulative change over time. Both phonemic and category fluency tasks reflect aspects of lexico-semantic processing and executive function (34, 35). In addition, a comprehensive battery of neuropsychological measures was performed at baseline as well as immediately, 6 weeks, and 14 weeks after completion of the 10 HD-tDCS treatment sessions. For cognitive outcome measures, we evaluated verbal retrieval function (phonemic fluency using FAS and category fluency using animal category, Boston naming test), executive functions (Trails Making B, Delis-Kaplan Executive Function Systems color-word interference), speed of processing (Trails Making A) and facial recognition (the Benton face test) (see references in Table 1). Electrophysiological measures (to be described in detail later) were also recorded at baseline, immediately, and at 6 weeks after completion of the 10 HD-tDCS sessions. We used different parallel versions as were available for HVLT-R and Digit Span for testing. Given limited versions available for any other tests, we did not use multiple versions.

**Table 1:** Neuropsychological test results.

	**Pre**	**Post- immediate**	**Post- 6 weeks**	**Post- 14 weeks**
Trails A^a^ – a sec (T)	123 (2)	37 (35)	28 (42)	29 (38)
Trails B^a^ - sec (T)	220 (11)	77 (34)	65 (37)	59 (42)
Digit Span^b^ - ss	9	11	11	13
Delis Kaplan Color Word Interference Test^c^				
Naming - sec (ss)	56 (1)	32 (8)	31 (8)	39 (5)
Reading - sec (ss)	19 (12)	19 (12)	19 (12)	23 (9)
Inhibition - sec (ss)	101 (1)	83 (2)	70 (6)	68 (6)
Inhibition-switching - sec (ss)	159 (1)	66 (8)	57 (10)	55 (11)
Phonemic fluency^d^ - items (T)	15 (19)	51 (51)	39 (40)	44 (44)
Category fluency^e^ – items (T)	20 (38)	29 (55)	24 (46)	24 (46)
Boston Naming Test^f^ - raw (T)	56 (39)	58 (51)	53 (31)	57 (43)
The Hopkins verbal learning test-revised^g^				
Total recall - raw (T)	28 (50)	30 (54)	31 (57)	29 (52)
Delayed recall – items, total of 12	10	12	12	11
Rey-Osterrieth complex figure^h^				
Copy - raw score (T)	36 (54)	36 (54)	36 (54)	36 (54)
Immediate recall - raw (T)	19.5 (44)	22 (49)	26 (57)	24 (53)
Delay recall - raw (T)	21 (47)	23 (51)	25 (55)	23.5 (52)
Benton Face^i^ - No. correct trials, total of 54	37	37	33	33

*Raw: raw scores; T: T scores; ss: scaled scores*.

a *Trails A&B: Trails Making Test (26)*.

b *Digit Span (27)*.

c *D-KEFS: Delis Kaplan Color Word Interference Test (28)*.

d *Phonemic fluency test: Controlled Oral Word Association Tests (COWAT) (29)*.

e *Category fluency: animal fluency (29)*.

f *BNT: Boston Naming Task (30)*.

g *HTLV-R: The Hopkins verbal learning test-revised (31)*.

h *Rey-O: Rey-Osterrieth complex figure (32)*.

i *Benton facial recognition test (33)*.

### HD-tDCS Effects After Each Single Session

After each session, we observed immediate improvements in verbal fluency (Figure 2B), but these changes did not persist until the next session.

### HD-tDCS Effects on Neuropsychological Measures After 10 Sessions

As shown in Table 1, at baseline, the patient exhibited impaired performance (<1 standard deviation from the average) in verbal retrieval (phonemic and category fluency, Boston naming test). She also demonstrated impaired executive functions (Trails Making B, color-word interference), speed of processing (Trails Making A) and facial recognition (the Benton face test) on neuropsychological measures (Figure 2C). Otherwise, she had near average performance in working memory, visuo-spatial and verbal learning functions (Table 1). Improvement in performance was found in tasks at which the patient was most impaired, most evident in verbal retrieval and executive function tests, including trails B, confrontational naming, phonemic and category fluency (Table 1, Figure 2C). These positive therapeutic effects persisted for at least 14 weeks after HD-tDCS was completed. In inhibition and inhibition/switch parts of the color-word interference test, we found incremental improvements over time, indicating delayed effects.

### HD-tDCS Effects on Electrophysiological Outcome Measures

**Figure 3:**
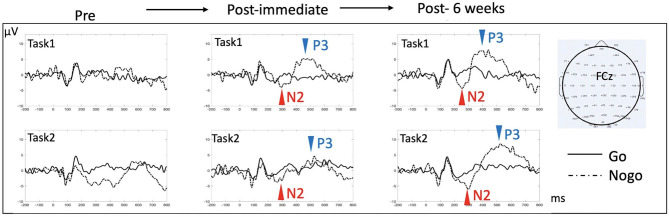
ERP effects in response to HD-tDCS. ERPs at the midline fronto-central electrode (FCz) were examined for the two response inhibition tasks (Go/NoGo), each contingent upon a different level of complexity [Task 1 is more perceptually based, requiring distinguishing between single exemplars of a car and a dog; Task 2 is more semantically based, requiring distinguishing between multiple exemplars of animals from non-animal objects. Refer to (36) for more detailed description of the tasks]. N2 (a negative deflection of evoked potentials peaking around 200 ms post-stimulus) and P3 (a positive deflection of evoked potentials peaking 300–500 ms post-stimulus) components are classically reported in this type of task, with NoGo trials eliciting more prominent N2/P3 amplitude than do Go trials. There was no evident N2/P3 prior to HD-tDCS. Both N2 and P3 components appeared after 10 sessions of HD-tDCS, which persisted after 6 weeks. In Task 1, comparing immediate post to 6 weeks post data, N2 peak amplitude/latency changed from −4.1 μV/293 ms to −4.4 μV/255 ms, while P3 peak amplitude/latency changed from 5.2 μV/448 ms to 7.7 μV/382 ms. In Task 2, N2 peak amplitude/latency changed from −3.8 μV/267 ms to −6.1 μV/298 ms, while P3 peak amplitude/latency changed from 4.6 μV/527 ms to 8.6 μV with a latency at 525 ms.

In order to investigate underlying neural changes in response to HD-tDCS intervention, we recorded EEG and evaluated N2/P3 event-related potentials (ERPs) components at baseline, immediately and at 6 weeks after completion of HD-tDCS treatment. EEG was recorded during two Go/NoGo tasks, including 2 different levels of perceptual and semantic complexity [applied extensively in a series of prior studies where more detailed description can be found, e.g., in (36)]. The N2 and P3 components during the response inhibition (Go/NoGo) paradigm are in general thought to represent correlates of cognitive control and response selection/inhibition (37, 38). While we recorded absent and markedly diminished N2/P3 components prior to HD-tDCS, we found restoration and normalization of the typical N2/P3 ERP components after completion of treatment (Figure 3), which persisted until 6 weeks after intervention, with overall increased amplitude and decreased latency in both N2 and P3 components. Behavioral performance improved significantly as well, with Go reaction time reduced from 635 ms at baseline to 447 and 327 ms immediately and at 6 weeks post intervention, respectively, in the more perceptually driven task (Figure 3, Task 1), and improved from 662 ms at baseline to 559 and 494 ms immediately and 6 weeks post intervention, respectively, in the more semantically driven task (Figure 3, Task 2). Accuracy remained relatively stable above 90% except for the baseline Go accuracy that was 77 % but improved to above 95% for all subsequent testing.

### Patient Report of HD-tDCS Effects

The patient reported significant and progressive improvement in her daily function during and after HD-tDCS. Before treatment she had dizziness that could be triggered by rapid head and eye movements. Her dizziness improved such that she started reading books, doing grocery shopping and watching her daughter's football practices. Second, she had trouble organizing and following her schedule and constantly relied on her notes and calendar. She noticed that after HD-tDCS she was able to follow plans by memory, and was less dependent on notes. Third, before she began this treatment regimen she experienced mental fatigue quite easily after limited duration of cognitive activity. After HD-tDCS, she had more energy and could carry out more daily tasks that she had not been able to prior to treatment.

## Discussions

In this TBI patient with baseline cognitive sequalae including persistent verbal retrieval and executive function deficits, we found clear responses to HD-tDCS intervention focused at the pre-SMA/dACC. We were able to show underlying neural correlates of her persistent behavioral changes.

By targeting the pre-SMA/dACC, we found improved confrontational naming suggesting improvement in verbal retrieval function, and improved verbal fluency, suggesting improvement in both verbal retrieval and executive functions. We also found improvements in those tasks especially involving the frontal lobes such as working memory, processing of speed, and executive functions, which plausibly reflect the potential role of the pre-SMA/dACC in these cognitive functions (12). The pre-SMA has been proposed to be involved in domain general processing regardless of stimulus modalities [for both motor and language selection, as in (9)] and its functional/structural connectivity to other pre-frontal regions as well as subcortical regions makes it a hub for higher cognitive processing including conflict resolution and cognitive control, as compared to more posterior regions such as the SMA proper and the primary motor cortices (11, 12). It is possible that some electric current spread to other frontal regions, effecting tDCS modulation in these regions, based on the simulated head model with the HD-tDCS montage (Figure 2). These would potentially explain the observed cross-domain/domain-general effects. Nevertheless, we did not find improvements in the impaired facial recognition, and her visuospatial and verbal episodic memory remained relatively stable despite HD-tDCS, supporting some level of response selectivity.

We also found variable durations of the HD-tDCS effects on performance of different types of tasks. Of note, tDCS effects have been proposed to reflect hormetic response, which simulates an inverse U-shape curve that only appears linear outside the accumulative dose range thought to be associated with the adaptive response (19). It is possible that the optimal hormetic response for each cognitive/motor function is different because of differences in function and sensitivity of underlying neural circuits. In general, short-term effects on neural function can be mediated through neuronal and synaptic activity (bottom-up processes), neuronal network dynamics (top-down processes), or a combination (39, 40). This hormetic response may also reflect long (er) term neuromodulatory effects mediated through either direct stimulation or a compensatory response to homeostatic perturbation, resulting in an adaptive response (19). The cumulative effect results from multiple repetitive HD-tDCS sessions (direct stimulation), while the adaptive responses even after completion of 10 HD-tDCS sessions (compensatory response) plausibly reflects ongoing effects. This latter effect may have been manifested by incremental improvements over time, even after HD-tDCS was completed, such as D-KEFS inhibition and inhibition-switch tests. A recent study using a similar HD-tDCS montage in veterans with TBI showed a delayed and extended improvement in category fluency that potentially supports this contention (15).

Aside from standardized neuropsychological measurements, the ERP findings provide strong evidence of the HD-tDCS modulatory effects on underlying neural circuits. Given that ERPs are thought to index both excitatory and inhibitory processes, the re-emergence of N2 and P3 components suggests HD-tDCS modulates synaptic potentials that underlie successful response selection and inhibition, supported also by improved Go trial reaction time. It has been shown that N2 and P3 components are generated from multiple brain regions with the most consistent generators from the frontal cortices such as the pre-SMA/dACC [(41); also based on Go/NoGo fMRI studies as in (42)]. Therefore, the observed modulation effect could potentially be mediated by either recruiting more neurons (that underly N2/P3 components) or bringing the underlying neuronal population into increased synchrony (16). These systems-level effects can be examined in the future with time-frequency power and phase coherence analysis.

We acknowledge that this is a single case study so the results may not be generalizable to other TBI patients and future research is warranted to include more patients to test treatment efficacy, which lies beyond the scope of the current report. One should take caution in designing and administering HD-tDCS in moderate to severe TBI populations with skull or brain lesions in that local current density could be enhanced over fissures or cranial penetrations (burr holes, etc) (25). Second, we did not have a sham condition and some improved performance could be related to learning/practice effect. However, the degree of improvement and the pattern of change during inter-trial testing (significantly better after each session rather than steady incremental change over sessions) would not be fully explained by learning effect. In addition, some impaired performance did not change significantly or to a similar extent after intervention (such as Benton face recognition and color-word interference inhibition part) as well as no changes on repeated assessment on tasks that that were initially intact, suggesting differential intervention effects but not an overall learning effect. Third, dizziness was a main complaint that affected the patient's daily activity and we did not assess how dizziness symptom might be associated with improved scores, although it is unlikely that improved performance was the result of improved dizziness. The patient did not complain of dizziness that limited her performance during neuropsychological testing.

## Conclusion

Our goal was to examine the therapeutic effects of HD-tDCS targeting the pre-SMA. While more systematic and randomized group studies will be needed to validate HD-tDCS as an effective treatment option, we demonstrated in this single patient the considerable potential that the technique has for addressing deficits in patients who have incurred TBI.

## Data Availability Statement

The original contributions presented in the study are included in the article/supplementary material, further inquiries can be directed to the corresponding authors.

## Ethics Statement

The studies involving human participants were reviewed and approved by the Institutional Review Board of the University of Texas at Dallas and the University of Texas Southwestern Medical Center. The patient/participant provided their written informed consent to participate in this study. Written informed consent was obtained from the participant for the publication of any potentially identifiable images or data included in this article.

## Author Contributions

H-SC designed and implemented the data analyses and wrote the manuscript. SS participated in data interpretation and analyses. JH and MK advised analysis framework. JH, MK, and SV designed the HD-tDCS protocol. All authors participated in editing.

## Conflict of Interest

The authors declare that the research was conducted in the absence of any commercial or financial relationships that could be construed as a potential conflict of interest.
