# Corrigendum: Case report: Improving verbal retrieval deficits with high definition transcranial direct current stimulation targeting the pre-supplementary motor area in a patient with chronic traumatic brain injury

**DOI:** 10.3389/fneur.2023.1177589

**Published:** 2023-03-14

**Authors:** Hsueh-Sheng Chiang, Scott Shakal, Sven Vanneste, Michael Kraut, John Hart

**Affiliations:** ^1^Department of Neurology, The University of Texas Southwestern Medical Center, Dallas, TX, United States; ^2^School of Behavioral and Brain Sciences, The University of Texas at Dallas, Richardson, TX, United States; ^3^Texas College of Osteopathic Medicine, The University of North Texas Health Science Center, Fort Worth, TX, United States; ^4^Trinity College Dublin, Dublin, Ireland; ^5^Department of Radiology and Radiological Science, The Johns Hopkins University School of Medicine, Baltimore, MD, United States

**Keywords:** TBI, verbal retrieval, HD-tDCS, EEG, case report, verbal fluency, tDCS, word finding

In the published article, there was an error. Some of the return electrodes were incorrectly described.

A correction has been made to “HD-tDCS Protocol and Outcome Measures,” paragraph 1, the first sentence. This sentence previously stated:

“The HD-tDCS montage targeting the pre-SMA consisted of Fz for the central anodal electrode and FP1, FP2, F7, and F8 for the return (cathodal) electrodes (i.e., five circular Ag/AgCl electrodes 1 cm radius with conductive gel).”

The corrected sentence appears below:

“The HD-tDCS montage targeting the pre-SMA consisted of Fz for the central anodal electrode and FPz, Cz, F7, and F8 for the return (cathodal) electrodes (i.e., five circular Ag/AgCl electrodes 1 cm radius with conductive gel).”

The authors apologize for this error and state that this does not change the scientific conclusions of the article in any way. The original article has been updated.

